# *JAK2*-mutant clonal hematopoiesis is associated with venous thromboembolism^∗^

**DOI:** 10.1182/blood.2024024187

**Published:** 2024-07-18

**Authors:** Rebecca L. Zon, Aswin Sekar, Katharine Clapham, Ohad Oren, Abhishek Niroula, Alexander G. Bick, Christopher J. Gibson, Gabriel Griffin, Md Mesbah Uddin, Donna Neuberg, Pradeep Natarajan, Benjamin L. Ebert

**Affiliations:** 1 Department of Medical Oncology, Dana-Farber Cancer Institute, Boston, MA; 2 Department of Medicine, Brigham and Women’s Hospital and Harvard Medical School, Boston, MA; 3 Broad Institute of Massachusetts Institute of Technology and Harvard, Cambridge, MA; 4 Department of Medicine, University of Utah School of Medicine, Salt Lake City, UT; 5 Cardiovascular Research Center and Center for Genomic Medicine, Massachusetts General Hospital and Harvard Medical School, Boston, MA; 6 Division of Cardiology, Massachusetts General Hospital, Boston, MA; 7 Institute of Biomedicine, SciLifeLab, University of Gothenburg, Gothenburg, Sweden; 8 Department of Medicine, Vanderbilt University Medical Center, Nashville, TN; 9 Department of Data Science, Dana-Farber Cancer Institute, Boston, MA; 10 Howard Hughes Medical Institute, Dana-Farber Cancer Institute, Boston, MA

## Abstract

Venous thromboembolism (VTE) is common among older individuals, but provoking factors are not identified in many cases. Patients with myeloid malignancies, especially myeloproliferative neoplasms (MPNs), are at increased risk for venous thrombosis. Clonal hematopoiesis of indeterminate potential (CHIP), a precursor state to myeloid malignancies, is common among older individuals and may similarly predispose to venous thrombosis. We evaluated overall and genotype-specific associations between CHIP and prevalent and incident VTE in >400 000 samples from the UK Biobank. CHIP was modestly associated with incident VTE with a hazard ratio (HR) of 1.17 (95% confidence interval [CI], 1.09-1.3; *P =* .002) but was not significantly associated with prevalent VTE with an odds ratio (OR) of 1.02 (95% CI, 0.81-1.23; *P =* .81). *TET2-*mutant CHIP was associated with incident VTE with a HR of 1.33 (95% CI, 1.05-1.69; *P =* .02). *JAK2* mutations were highly associated with both prevalent and incident VTE risk, with an OR of 6.58 (95% CI, 2.65-16.29; *P =* 4.7 × 10^–5^) and a HR of 4.2 (95% CI, 2.18-8.08; *P =* 1.7 × 10^–5^), respectively, consistent with the thrombophilia associated with *JAK2*-mutant MPN. The association between *JAK2-*mutant CHIP and VTE remained significant after excluding potential undiagnosed MPN based on laboratory parameters. *JAK2-*mutant CHIP was more strongly associated with VTE but was less common than heterozygous factor V Leiden and heterozygous prothrombin gene mutation. These results indicate that most individuals with CHIP do not have an altered risk of thrombosis, but individuals with *JAK2*-mutant CHIP have a significantly elevated risk of VTE.

## Key Points

• *JAK2*-mutant clonal hematopoiesis is associated with a strong risk of VTE.
• *JAK2-*mutant CHIP confers a greater risk of VTE than heterozygous thrombophilia but is present at a lower frequency in the general population.

## Visual Abstract

**Figure:**
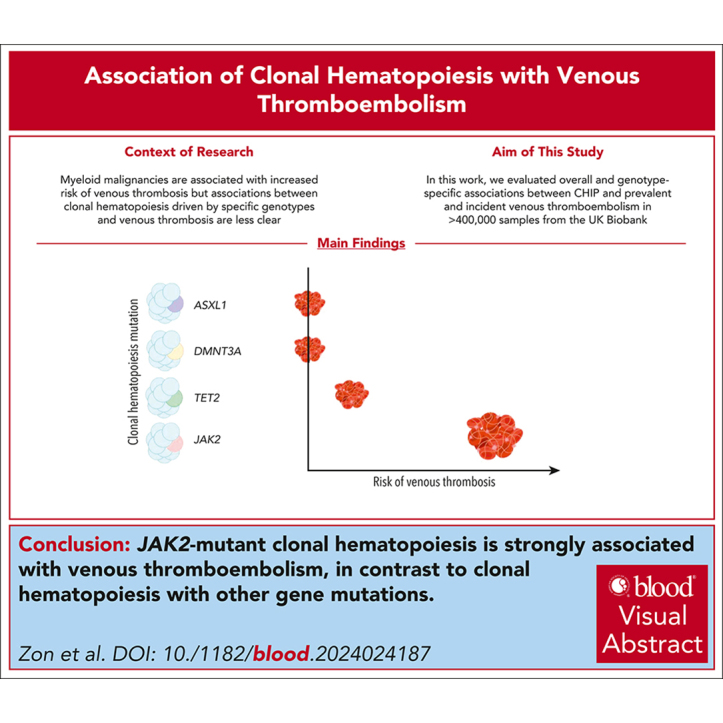

## Teaser

An association between clonal hematopoiesis of indeterminate potential (CHIP) and venous thromboembolism (VTE) has been suggested in epidemiological studies. Zon et al evaluated CHIP genotype–specific associations with VTE in a very large general population data and sample set from the UK Biobank. After accounting for potentially undiagnosed myeloproliferative diseases, the authors established that *JAK2*-mutant CHIP was highly associated with risk of VTE, whereas *TET2*-mutated CHIP was only modestly associated; *DNMT3A*-mutated CHIP, the most common genotype, was unassociated with increased VTE risk.

## Introduction

Venous thromboembolism (VTE) affects nearly 10 million people worldwide every year.^1^ Risk factors for VTE include recent major surgery, malignancy, and inherited thrombophilia. However, 25% to 50% of venous thromboembolic events do not have a clear, identifiable cause.^2^ Myeloid malignancies increase the risk of venous thrombosis, especially among those with myeloproliferative neoplasms (MPNs).^3^^,^^4^ Studies have shown a 10-fold increased risk of MPN-associated thrombotic events compared with healthy individuals.^5^, ^6^, ^7^

Clonal hematopoiesis of indeterminate potential (CHIP) is a premalignant state characterized by myeloid malignancy driver mutations with a variant allele fraction ≥0.02 in individuals without blood count abnormalities.^8^^,^^9^ CHIP increases in prevalence with age, occurring in ∼10% of individuals aged >70 years.^10^^,^^11^ CHIP is associated with an increased risk of hematologic malignancies and has also been associated with nonhematologic pathologies. These conditions, many of which are etiologically linked to inflammation, include cardiovascular disease,^12^^,^^13^ stroke,^14^ kidney disease,^15^^,^^16^ rheumatologic conditions,^17^ and liver disease.^18^

Studies evaluating the relationship between CHIP and VTE have arrived at conflicting results. Retrospective studies have found prevalent CHIP in 19% of patients with unprovoked pulmonary embolism,^19^ 37.8% and 46% in patients with splanchnic vein thrombosis,^20^^,^^21^ and 3.7% in patients with unprovoked proximal VTE.^22^ In a larger cohort study, in which 45% of individuals had schizophrenia, incident venous thrombosis rates in patients with CHIP were 5%, compared with 2.1% in patients without CHIP.^23^ Examination of genotype-specific associations between CHIP and VTE requires substantially larger cohorts. We, therefore, examined CHIP and VTE in the UK Biobank, a study with >400 000 individuals linked to exome sequencing of peripheral blood DNA and detailed clinical phenotypes.

## Methods

### UK Biobank and analyses

A total of 502 490 individuals from the UK Biobank were evaluated for inclusion in this study. Data were obtained for these participants through UK Biobank (application number 50834). Individuals with whole-exome sequencing data and nonmissing data across all covariates were included. Individuals were excluded if genomic analyses did not pass quality control, individuals were outliers for heterozygosity or missing rate, the individual had a prevalent diagnosis of a hematologic malignancy (list of diagnosis codes as previously described^24^) before DNA sampling or within 6 months after enrollment in the study, or consent was withdrawn.

VTE events were identified based on International Classification of Diseases (ICD10) codes (supplemental Table 1, available on the *Blood* website). Prevalent and incident VTE cases were ascertained with respect to the time of DNA sampling. Prevalent VTE was defined as having a VTE at or before the time of DNA sampling. An incident VTE was defined as an event that happened after DNA sampling. Median follow-up time for incident VTE was 11.8 years. Associations of CHIP and VTE were assessed using logistic regression for prevalent analyses and Cox proportional hazards for incident analyses. For the incident analysis, we excluded those with prevalent VTE at the time of DNA sampling. The association analyses were adjusted for age at the time of DNA sampling, age^2^, sex, European ancestry, first 5 genetic principal components, smoking status (ever vs never), and body mass index as covariates. Analyses were conducted in R Studio.

### CHIP detection

CHIP mutations were identified as described previously^24^ using whole-exome sequencing data from blood DNA from UK Biobank participants. Somatic variants in genes associated with clonal hematopoiesis and/or myeloid malignancies were detected using Mutect2.^10^^,^^25^, ^26^, ^27^ The list of genes in which pathogenic mutations were identified for CHIP calling, and gene-level sequencing coverage statistics for this cohort are described elsewhere.^28^ The variant allele fraction had to be at least 0.02 to be considered a CHIP mutation. A minimum total read depth of 20 and a minimum read depth of 5 supporting the alternative allele were required. To minimize likelihood of detecting germ line variants and artifacts, the Genome Aggregation Database was used as a germ line reference, and a panel-of-normal derived from the youngest participants in the UK Biobank cohort was used.

### Definition of potential undiagnosed cases of MPN

With the inclusion of a *JAK2* mutation, laboratory findings suggestive of MPN were defined as polycythemia vera with hemoglobin >16.5 g/dL for males or hemoglobin >16 g/dL for females or essential thrombocytosis with platelets >450 × 10^9^/L based on the International Consensus Classification.^29^ *JAK2* mutations in this analysis were all V617F.

### Association of VTE with heterozygous inherited thrombophilias

Association of VTE with factor V Leiden (heterozygous) and prothrombin gene mutation (heterozygous) was analyzed in the UK Biobank based on single nucleotide polymorphism genotypes for rs6025 and rs1799963, respectively. The risk associated with heterozygous factor V Leiden or prothrombin gene mutation was compared with that of individuals who were homozygous wild type (individuals with homozygous factor V Leiden mutation or homozygous prothrombin mutation were not included in the analysis given their low frequency). Association was analyzed in a logistic regression model with VTE as the binary outcome variable and single nucleotide polymorphism genotype as the predictor variable with covariates of age, age^2^, sex, body mass index, European ancestry, smoking status (ever vs never), and the first 5 genetic principal components as covariates.

## Results

### Analysis of CHIP with incident VTE

Given the established association between myeloid neoplasms and VTE, we sought to determine whether a similar relationship existed between CHIP and VTE. Of 502 490 individuals in the UK Biobank, 425 399 individuals had high-quality exome sequencing, did not have prevalent hematologic malignancies by ICD10 codes, and had documented covariates. Among these individuals, the mean age of the participants was 56.6 years, 54% were female, 84% were of European ancestry, and 60% had ever smoked.

**Figure 1.**
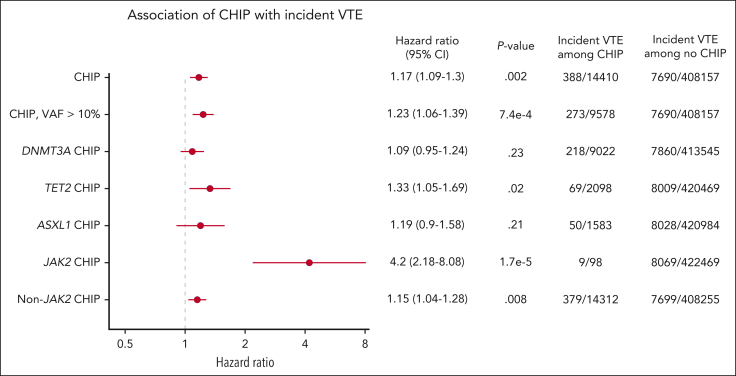
**Incidence of VTE in individuals with and without CHIP analyzed using Cox proportional hazards, corrected for age, sex, European ancestry, genetic principal components, ever smoked status, and body mass index.**

CHIP was modestly associated with incident VTE with a hazard ratio (HR) of 1.17 (95% confidence interval [CI], 1.09-1.3; *P =* .002), and CHIP with variant allele fraction (VAF) >10% had a HR of 1.23 (95% CI, 1.06-1.39; *P =* 7.4 × 10^–4^; Figure 1). We evaluated the 3 most commonly mutated genes in CHIP and found that *TET2*-mutant CHIP was modestly associated with incident VTE, with a HR of 1.33 (95% CI, 1.05-1.69; *P =* .02), but *DNMT3A*-mutant and *ASXL1*-mutant CHIP were not significantly associated with VTE.

Because *JAK2*-mutant MPNs are a potent risk factor for VTE, we hypothesized that *JAK2*-mutant CHIP may be particularly associated with an increased risk of VTE. Indeed, we found that *JAK2*-mutant CHIP was also strongly associated with incident VTE with a HR of 4.2 (95% CI, 2.18-8.08; *P =* 1.7 × 10^–5^). Among 98 individuals with *JAK2*-mutant CHIP, 9 had a documented incident VTE during a median of 6.98 years of follow-up (Figure 1). Given the strong association between *JAK2*-mutant CHIP and VTE and the fact that patients with *JAK2-*mutant MPNs have been found to have venous thrombotic events at unusual sites, such as splanchnic vein thromboses,^30^^,^^31^ we evaluated the types of venous thrombotic events seen in our cohort. In the 9 individuals with *JAK2*-mutant and incident VTE, 4 had deep vein thrombosis, 4 had pulmonary embolism, and 1 had portal vein thrombosis. In these 9 individuals, 6 developed an MPN after DNA sampling and before or at the time of incident VTE.

### *JAK2-*mutant CHIP is strongly associated with prevalent VTE

**Figure 2.**
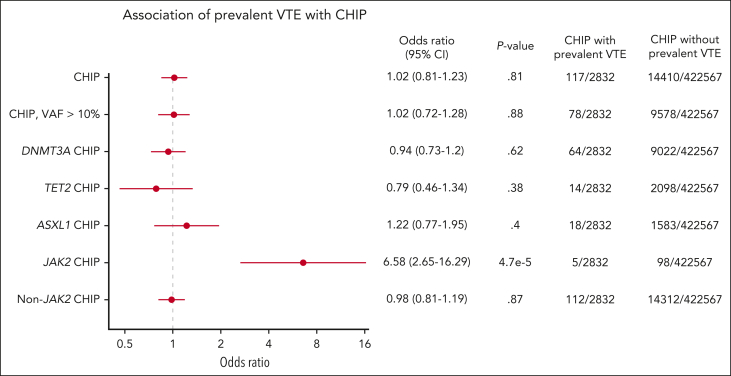
**Prevalence of CHIP in individuals with and without VTE analyzed using logistic regression, corrected for age, sex, European ancestry, genetic principal components, ever smoked status, and body mass index.**

Because CHIP may exist for many years, we analyzed the frequency of CHIP in individuals with and without prevalent VTE (defined as a VTE event at or before DNA sampling). We again found that *JAK2*-mutant CHIP was strongly associated with prevalent VTE, with an odds ratio (OR) of 6.58 (95% CI, 2.65-16.29; *P =* 4.7 × 10^–5^). Among 2832 individuals with a prevalent VTE, 5 individuals had *JAK2* mutations. Of these 5 individuals, 2 had deep vein thrombosis, 1 had pulmonary embolism, 1 had portal vein thrombosis, and 1 had Budd Chiari based on ICD10 codes. In contrast, there was no association found between prevalent VTE and CHIP overall (OR, 1.02; 95% CI, 0.81-1.23; *P =* .81) or CHIP overall with VAF >10% (OR, 1.02; 95% CI, 0.72-1.28; *P* = .88; Figure 2).

### *JAK2*-mutant CHIP is strongly associated with VTE after excluding potential undiagnosed MPNs

**Figure 3.**
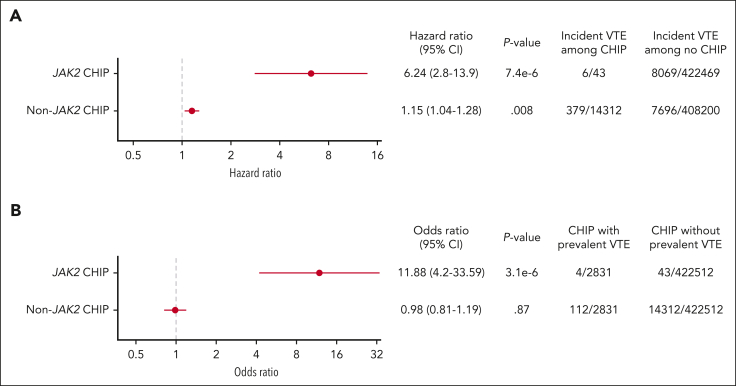
**Association between (A) *JAK2-*mutant CHIP and incident VTE and (B) *JAK2-*mutant CHIP and prevalent VTE with myeloid neoplasms excluded by ICD10 codes and potential cases of undiagnosed MPN excluded based on cytoses.**

We next considered the possibility that the VTE association with *JAK2*-mutant CHIP could be driven by undiagnosed cases of MPNs that are not diagnosed or captured by ICD10 codes. To identify potential *JAK2*-mutated MPNs in the UK Biobank that were not identified based on ICD10 codes, we analyzed the elevations in laboratory values of hemoglobin and platelet counts using criteria established by the International Consensus Classification (supplemental Table 2).^9^ With MPN diagnoses excluded by both ICD10 codes and in those with cytoses, *JAK2*-mutant CHIP remained significantly associated with incident VTE with a HR of 6.24 and prevalent VTE with an OR of 11.88 (Figure 3A-B, respectively). Although those with cytopenias may represent advanced disease and would therefore be more likely to have a formal diagnosis, we further analyzed the association of *JAK2*-mutant CHIP with both incident and prevalent VTE when MPN diagnoses were excluded based on ICD10 codes, cytoses, and cytopenias; the HR for incident VTE with *JAK2-*mutant CHIP was 7.49, and the prevalent odds for VTE ratio was 14.59 (supplemental Figure 1).

### Risk of VTE with *JAK2-*mutant CHIP relative to inherited heterozygous thrombophilias

Although *JAK2*-mutant CHIP was associated with a significantly increased risk of incident VTE (HR, 6.24), it accounted for only a small fraction (5/2832) of individuals with prevalent VTE (Table 1). The median follow-up time to VTE for *JAK2-*mutant CHIP was 6.98 years and for non-*JAK2-*mutant CHIP was 7.23 years. To contextualize these findings with well-established risk factors for VTE in the same cohort, we examined the risk of inherited thrombophilias. Using germ line genetic data in the UK Biobank, we identified individuals with factor V Leiden and prothrombin gene mutation. Heterozygous factor V Leiden and heterozygous prothrombin gene mutation G20210A had HRs of 2.36 and 1.91, respectively. The median age of VTE diagnosis for both heterozygous thrombophilias was 64.88 years. These studies demonstrate that the impact of *JAK2*-mutant CHIP on the risk of VTE may be greater than common heterozygous inherited thrombophilias, although the prevalence of *JAK2*-mutant CHIP is lower than these germ line risk alleles.

**Table 1.** ***JAK2-*mutant CHIP relative incident risk of VTE and prevalence in the general population compared with heterozygous factor V Leiden and heterozygous prothrombin gene mutation G20210A in the UK Biobank**

Thrombophilia	HR of first episode of VTE compared with controls	Prevalence in the population
Heterozygous factor V Leiden	2.36 (95% CI, 2.21-2.52)	4.4%
Heterozygous prothrombin G20210A	1.91 (95% CI, 1.74-2.10)	2.3%
*JAK2* CHIP	6.24 (95% CI, 2.8-13.9)	98/430 000 (0.02%) 0.14%-3% in other studies^11^^,^^32^, ^33^, ^34^
Non-*JAK2* CHIP	1.15 (95% CI, 1.04-1.28)	14 417/430 000 (3.4%)

## Discussion

Our study demonstrates definitively that, in a large population-based cohort with whole-exome sequencing, *JAK2*-mutant CHIP is most strongly associated with both incident and prevalent VTE compared with other CHIP genotypes. Although other CHIP genotypes have been associated with inflammatory disorders, VTE is selectively associated with *JAK2* mutations.^10^^,^^12^^,^^35^^,^^36^ Our findings validate reports from smaller cohorts, nonpopulation-based cohorts, or cohorts that had more limited genotyping of somatic mutations.^23^^,^^37^, ^38^, ^39^ The relationship between *JAK2*-mutant CHIP and VTE likely involves multiple mechanisms including increased release of neutrophil extracellular traps and impacts on platelets and red blood cells.^23^^,^^32^ *JAK2*-mutant CHIP was also recently found to increase the risk of arterial thrombosis due to younger and more activated platelets.^33^

The UK Biobank cohort enabled us to examine the strength of association between *JAK2*-mutant CHIP and VTE, as well as the impact of germ line predisposition to VTE in the same population. Similarly, the relative prevalence of these germ line and somatic risk factors was assessed in the same cohort. Somatic *JAK2* mutations carry a higher risk of thrombosis in the UK Biobank than inherited mutations in the factor V or prothrombin genes. On the contrary, somatic *JAK2* mutations were much rarer than the major inherited thrombophilias. The prevalence of *JAK2*-mutant CHIP in our study was 0.02%, which is lower than prior documented rates ranging from 0.14% to 3.1% depending on the depth of sequencing coverage.^11^^,^^37^, ^38^, ^39^ The exome sequencing data in the UK Biobank had lower coverage of the *JAK2* locus than other CHIP genes, thereby decreasing the sensitivity of detection for smaller *JAK2*-mutant clones. It is likely that the VTE risk from smaller *JAK2* clones is lower than the risk associated with larger clones, as has been demonstrated for other CHIP-associated phenotypes.^10^^,^^12^^,^^34^ A Danish population study of ∼20 000 individuals showed an OR for prevalent VTE of 2.8 in individuals with *JAK2* VAF ≥0.01 vs an OR of 0.71 with *JAK2* VAF <0.01, further supporting the increased risk of VTE with larger *JAK2* clone sizes as found in our cohort.^38^ Using an estimated prevalence of *JAK2* V617F of 2 of 1000 in the general population based on the middle of the range from our study and others^11^^,^^37^, ^38^, ^39^ and ∼77 million individuals aged >60 years in the United States,^40^ 154 000 individuals in the United States would be estimated to have *JAK2*-mutant CHIP.^34^

Our findings demonstrate that, among individuals with CHIP, those with *JAK2*-mutant CHIP have a striking risk of developing a VTE. Although we do not recommend screening for *JAK2* mutations, particularly due to the low prevalence of *JAK2*-mutant CHIP, the index of suspicion may be increased by blood count parameters or VTE location. In the UK Biobank, individuals with CHIP who developed a VTE event tended to have higher hemoglobin and/or platelet counts, even though values were within the normal range and would therefore not be consistent with a diagnosis of MPN based on these parameters. In addition, individuals with *JAK2*-mutant CHIP may have VTE events in unusual locations, such as splanchnic vein thrombosis, based on data from the UK Biobank and prior publications.^41^^,^^42^ Even without screening, widespread use of next-generation sequencing panels has resulted in an increased number of individuals diagnosed with *JAK2*-mutant CHIP. Future studies will aid in determining approaches to VTE risk reduction for individuals with *JAK2*-mutant CHIP.

Conflict-of-interest disclosure: B.L.E. has received research funding from Celgene, Deerfield, Novartis, and Calico; has received consulting fees from AbbVie and GRAIL; and is a member of the scientific advisory board and shareholder for Neomorph Inc, TenSixteen Bio, Skyhawk Therapeutics, and Exo Therapeutics. P.N. reports research grants from Allelica, Apple, Amgen, Boston Scientific, Genentech/Roche, and Novartis; personal fees from Allelica, Apple, AstraZeneca, Blackstone Life Sciences, Creative Education Concepts, CRISPR Therapeutics, Eli Lilly & Co, Esperion Therapeutics, Foresite Labs, Genentech/Roche, GV, HeartFlow, Magnet Biomedicine, Merck, Novartis, TenSixteen Bio, and Tourmaline Bio; equity in Bolt, Candela, Mercury, MyOme, Parameter Health, Preciseli, and TenSixteen Bio; and spousal employment at Vertex Pharmaceuticals, all unrelated to the present work. A.B. is on the scientific advisory board membership at TenSixteen Bio. K.C. is on the advisory board for United Therapeutics and receives consulting fees from Amgen, Tectonic Therapeutic, and United Therapeutics. A.S. reports stock in Vertex. R.L.Z. is a stockholder in and receives consultancy fees from Triveni Bio. The remaining authors declare no competing financial interests.
