# Supplementary material for: JAK2-mutant clonal hematopoiesis is associated with venous thromboembolism
Source: Blood. 2024 Jul 18;144(20):2149–54. doi: 10.1182/blood.2024024187 (PMC11600088; doi:10.1182/blood.2024024187)
Supplement: Supplemental Tables and Figure [file BLOOD_BLD-2024-024187-mmc1.pdf]

Supplemental Material

ICD10 Code	Number of individuals per ICD10 code
I26.0 Pulmonary embolism with mention of acute cor pulmonale	128/425399
I26.9 Pulmonary embolism without mention of acute cor pulmonale	6190/425399
I67.6 Nonpyogenic thrombosis of intracranial venous system	12/425399
I63.6 Cerebral infarction due to cerebral venous thrombosis, nonpyogenic	8/425399
I80.1 Phlebitis and thrombophlebitis of femoral vein	413/425399
I80.2 Phlebitis and thrombophlebitis of other deep vessels of lower extremities	3742/425399
I81 Portal vein thrombosis	292/425399
I82.0 Budd-Chiari syndrome	15/425399
I82.2 Embolism and thrombosis of vena cava	78/425399
I82.3 Embolism and thrombosis of renal vein	42/425399

Supplementary Table 1. Venous thromboembolism ICD10 codes.

Pt #	Sex	Age (years)	Hemoglobin (g/dL)	Platelets ($x10^9$/L)	VTE Location
1	F	49	14.6	578	PVT
2	F	71	13.3	560	DVT
3	M	61	20.3	479	DVT
4	F	75	15.1	276	DVT
5	F	72	14.4	305	PE
6	M	74	15.0	308	PE
7	F	73	15.8	259	DVT
8	M	77	14.4	385	PE
9	M	72	14.4	256	PE
10	F	67	16.1	488	PE
11	M	60	15.5	252	DVT
12	M	68	15.5	315	DVT
13	F	52	14.0	226	PVT
14	M	44	14.5	190	Budd Chiari syndrome

Supplementary Table 2. *JAK2*-mutant CHIP patient characteristics based on age, sex, laboratory values, and location of venous thromboembolism (VTE). Patients 1-9 (green) are from the incident VTE analysis and 10-14 (blue) are from the prevalent VTE analysis. Patients who were excluded due to meeting the laboratory value criteria for myeloproliferative neoplasm (hemoglobin >16 g/dL for females or essential thrombocytosis with platelets >450 x 10^9/L) are highlighted in yellow.
PVT= portal vein thrombosis: DVT= deep vein thrombosis; PE= pulmonary embolism

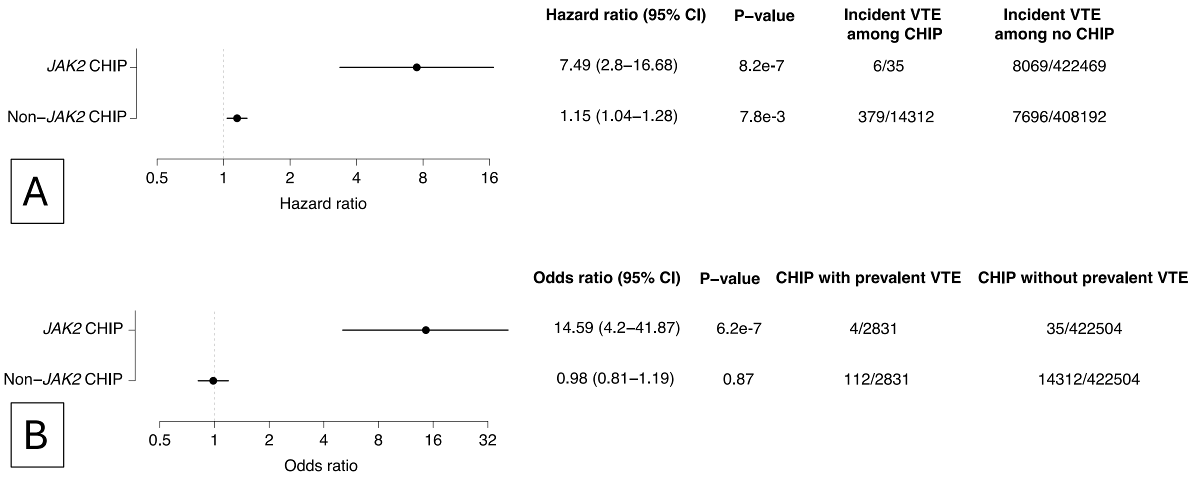

Supplementary Figure 1. Association between *JAK2*-mutant CHIP and incident VTE (A) and *JAK2*-mutant CHIP and prevalent VTE (B) with myeloid neoplasms excluded by ICD10 codes and potential cases of undiagnosed myeloproliferative neoplasms excluded based on cytoses and cytopenias.
